# Nonaplex PCR using Cliffhanger primers to identify diarrhoeagenic *Escherichia coli* from crude lysates of human faecal samples

**DOI:** 10.1371/journal.pone.0199766

**Published:** 2018-06-26

**Authors:** Uffe Vest Schneider, Nikolaj Dam Mikkelsen, Flemming Scheutz, Alice Friis-Møller, Gorm Lisby

**Affiliations:** 1 Anapa Biotech A/S, Hørsholm, Denmark; 2 Department of Clinical Microbiology, Copenhagen University Hospital Hvidovre, Denmark; 3 Statens Serum Institut, Copenhagen, Denmark; 4 The International Collaborating Centre for Reference and Research on *Escherichia* and *Klebsiella*, Department of Bacteria, Parasites and Fungi, Statens Serum Institut, Copenhagen, Denmark; University of Helsinki, FINLAND

## Abstract

Sensitive, probe-based detection of multiple DNA targets is limited by the competitive reannealing of the antiparallel duplex DNA helix with the complementary DNA strand. To address this, we developed Cliffhanger primers, which create single-stranded DNA overhangs on PCR amplicons while simultaneously increasing the multiplex PCR efficacy and allowing PCR amplification using crude lysates of human faecal samples. A multiplex PCR that targeted eight genes from diarrhoeagenic *Escherichia coli* plus an internal control was performed and compared to a routine method that consisted of culture followed by multiplex PCR with fragment length separation. A total of 2515 clinical faecal samples from patients with diarrhoea were tested using both methods, and there was a significant increase in clinical sensitivity and negative predictive value with the Cliffhanger method for seven out of eight genes. All Cliffhanger-only positive samples were confirmed by Sanger sequencing of the PCR amplicon. Notably, the Cliffhanger method reduced the total sample turn-around time in the laboratory from 20 hours to 6 hours. Hence, use of Cliffhanger primers increased assay robustness, decreased turn-around time and increased PCR efficacy. This increased the overall clinical sensitivity without the loss of specificity for a heavily multiplexed PCR assay.

## Introduction

Diarrhoeagenic *Escherichia coli* (DEC) causes both severe diarrhoea in children and travel-associated diarrhoea, and it represents an increasing burden on healthcare systems worldwide [1]. DEC can be divided into six categories based on the virulence characteristics: enteropathogenic *E*. *coli* (EPEC; *eae*-positive and a given serotype); enterotoxigenic *E*. *coli* (ETEC; *elt-*, *estAh-* or *estAp*-positive); diffusely adherent *E*. *coli* (DAEC); Enterohaemorrhagic *E*. *coli* (EHEC including the subgroup of Shiga toxin producing *E*. *coli*, STEC; *stx1-* or *stx2*-positive and a certain serotype); enteroinvasive *E*. *coli* (EIEC or *Shigella spp*; *ipaH-*positive); and enteroaggregative *E*. *coli* (EAEC; primarily a*ggR*-positive) [2,3,4].

When the clinical samples for this study were collected (from December 1, 2012 to March 20, 2013), the Department of Clinical Microbiology at Hvidovre University Hospital, Copenhagen routinely detected DEC by culturing samples on general enteric media. Three or four colonies were then picked and pooled, followed by multiplex PCR with fragment length separation. When the pooled colonies tested positive for the *stx1*, *stx2*, *ipaH* and/or *eae* genes, the individual colonies were picked a second time, and positivity was confirmed using the same PCR as for testing the pooled colonies. Subsequently, positive colonies were O-typed by agglutination to determine the pathogenicity of the isolate [3,5,6,7]. This method is laborious and time consuming, however, and it only detects DEC genes that are present in the selected *E*. *coli* colonies. At the time of the study, the Department of Clinical Microbiology at Hvidovre Hospital, Copenhagen routinely used selective media for detection of EHEC O157:H7 [8].

After the study began, several commercial assays for detecting DEC by multiplex PCR became available, but they remain expensive, which will likely limit the market penetration [9,10]. It would be ideal to directly detect the DEC genes and analyse all of the *E*. *coli* DNA present in faecal samples while also decreasing the diagnostic turn-around time. The challenge with this approach is that faecal samples have human and microbial DNA that essentially act to dilute the *E*. *coli* DNA with the genes; in addition, human faeces contain numerous PCR inhibitors [11].

**Fig 1:**
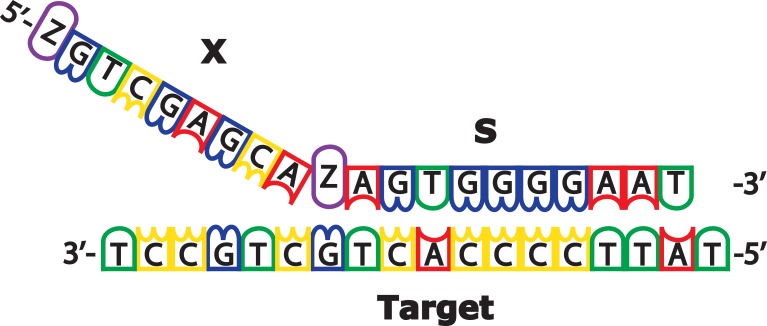
A Cliffhanger PCR primer with a Z-X-Z-S structure. Z is an *ortho*-TINA molecule, X is the custom-designed single-stranded nucleic acid overhang and S is the target-specific priming sequence. The 5’ *ortho*-TINA molecule protects the oligonucleotide against 5’ to 3’ exonuclease activity, whereas the internally placed *ortho*-TINA molecule blocks the DNA polymerase and thereby creates a single-stranded DNA overhang on the PCR amplicon.

*Ortho*-twisted intercalating nucleic acid (*o*-TINA) is a nucleic acid analogue that can improve the efficiency and robustness of multiplex PCR when added at the 5´-position of PCR primers [12]. The large aromatic *o*-TINA monomer blocks DNA polymerases, thus allowing the synthesis of PCR amplicons with single-stranded custom-designed overhang(s) when one or both PCR primer(s) are designed as Cliffhanger primers i.e. as Z-X-Z-S, where X is the custom-designed overhang sequence, Z is the *o*-TINA molecule and S is the specific priming sequence (Fig 1). The 5´ placement of a second *o*-TINA monomer also protects the PCR primers and amplicons in a PCR reaction against 5’ to 3’ exonuclease degradation.

The creation of single-stranded overhangs on PCR products enables the simultaneous detection of multiple targets that are amplified in a single reaction using hybridization-based solid-phase capture platforms, e.g. xTAG from Luminex, nanospheres and DNA chips. Solid-phase capture platforms can easily detect more than ten targets, surpassing the current limit of six targets using real-time PCR platforms. Furthermore, the single-stranded overhang on the PCR amplicons enables direct post-PCR detection of the amplified targets using a solid-phase capture platform without prior purification and denaturation of the PCR amplicons.

**Fig 2:**
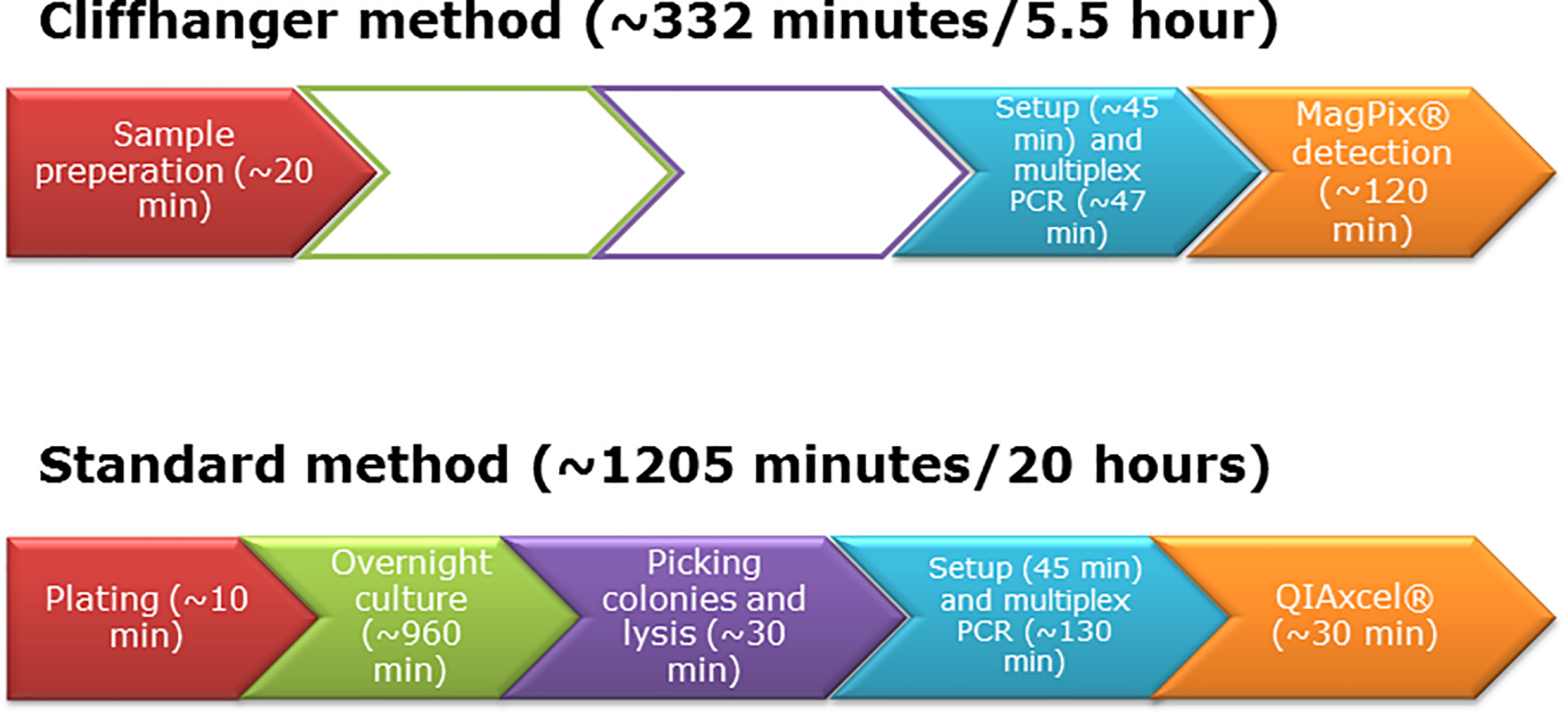
Workflows of the routine analysis method versus the Cliffhanger method.

The present work describes the design and construction of a highly multiplexed PCR assay for simultaneous amplification of eight genes (*aggR*, *eae*, *elt*, *estAh*, *estAp*, *ipaH*, *stx1* and *stx2*) that are used to identify DEC in crude lysates of human faecal samples. The *E*. *coli rrs* gene was used as an internal sample control. PCR was followed by direct detection without prior purification or denaturation of PCR amplicons on the Luminex platform. We compared the performance of the Cliffhanger method for analysing clinical samples from patients with diarrhoea versus the routine method, which consisted of culture followed by multiplex PCR with fragment length separation (Fig 2).

## Materials and methods

### Ethics statement

Danish National Committee on Health Research Ethics reviewed the proposed study and stated that no formal ethical approval was needed as the research was conducted as a method validation study. The clinical samples were examined by the routine method, and anonymized waste materials were then re-examined by the Cliffhanger method for validation prior to disposal.

### Clinical specimens

All clinical specimens received for DEC analysis at the Department of Clinical Microbiology at Copenhagen University Hospital Hvidovre between December 1, 2012 and March 20, 2013 were included in the study. A total of 2515 faecal specimens were included. Of these, 2005 specimens were collected into 2 mL of FecalSwab Cary-Blair Collection and Transport media (Copan Italia S.P.A., Brescia, Italia), and 510 specimens were collected as faecal material into dry containers (SSI Diagnostica, Hillerød, Denmark). All specimens were examined by the routine method at the Department of Clinical Microbiology and subsequently by the Cliffhanger method by Anapa Biotech. For each of the 510 specimens that were collected into dry containers, a 5 μL inoculation loop of faecal material was transferred to FecalSwab medium prior to sample preparation by Anapa Biotech.

### Sample preparation

For the routine method, a 5 μL inoculation loop of faecal material from either FecalSwab media or a dry container was plated onto an SSI Enteric plate to detect Enterobacteriaceae (article no. 22880, SSI Diagnostica, Hillerød, Denmark) by three-point spreading, and the plate was incubated overnight at 37°C in ambient air. The indicator medium in the enteric plate allows for growth differentiation and bacterial selection so that it differentiates between different genera within Enterobacteriaceae. The next day, duplicates of one to ten (four on average) morphologically distinct *E*. *coli* colonies were picked by trained laboratory technicians with a 1 μL inoculation loop and transferred to a 0.2 mL Eppendorf tube containing 100 μL sterile water. Each vial was incubated at 95°C for 15 minutes in a water bath prior to use.

For the Cliffhanger procedure, 10% (200 μL) of the initial 2 mL sampling volume of the FecalSwab specimen was transferred to a 2.0 mL Eppendorf tube and 22 μL of 2M NaOH was added. The mixture was vortexed briefly and then incubated at 95°C at 600 rpm for 5 minutes in a ThermoMixer. The faecal lysate was centrifuged for one minute at 10,000 rpm in an Eppendorf MiniSpin table centrifuge (Centrifuge Mixer Mini-01). Next, 150 μL of supernatant was transferred to a 1.5 mL Eppendorf tube containing 60 μL of 5 M (NH_4_)Ac, pH 7.5, and the sample was mixed for 30 seconds on level 3 in the Eppendorf MiniSpin table centrifuge. The lysates were stored at -65°C until further processing.

All samples were prepared for analysis by the Cliffhanger method within 24 hours of being plated for analysis by the routine method. The stored lysate specimens were processed by the Cliffhanger method within one month after the initial sample preparation.

### Bacterial controls and the DEC control collection

Three positive controls were prepared by mixing 30 colonies of clinical DEC strains into 6 mL of FecalSwab transport media (Copan Italia S.P.A, Brescia, Italia). Next, 200 μL of each control was aliquoted into separate 2.0 mL Eppendorf tubes and stored at -65°C until use. Each control was processed as described in the sample preparation section. The first control consisted of two clinical isolates: isolate 55989, which is an EAEC that is *aggR-*positive and *rrs*-positive; and D2435, which is an STEC that is *stx1-*, *stx2-* and *rrs-* positive. The second control was clinical isolate D2262, which is an ETEC that is *estAh-*, *estAp-*, *elt-* and *rrs*-positive. The third control consisted of two clinical isolates: D1826, which is an EPEC that is *eae-* and *rrs*-positive, and fr1368, which is an EIEC that is *ipaH-* and *rrs*-positive. The negative control was FecalSwab transport media that was inoculated with clinical isolate 9997, which is an *Enterococcus faecalis* isolate that is negative for all eight genes and *rrs*, as the primers are designed to be specific for *E*. *coli*.

All controls are clinical isolates that were collected in our laboratory or that were supplied by the International Collaborating Centre for Reference and Research on *Escherichia* and *Klebsiella*, Department of Bacteria, Parasites and Fungi at SSI. Each control was positive for two to three genes plus the internal *rrs* control. Accordingly, each positive control should be negative for five or six target genes, so it could be used to verify that non-specific amplification has not taken place in the multiplex PCR, but also ensures that non-specific hybridisation to the MagPlex microspheres has not taken place. Examples of median fluorescence intensity (MFI) for positive and negative controls on different plates are reported in S1 Table.

A clinical collection of 105 DEC reference strains was used to test both the routine method and the Cliffhanger method. DEC controls were plated on SSI Enteric plates by three-point spreading and incubated overnight at 37°C in ambient air. One colony was picked with a 1 μL inoculation loop, transferred to a 1.5 ml Eppendorf tube containing 100 μL of sterile water and incubated at 95°C for 15 minutes in a water bath prior to use.

### Primer design and validation

All oligonucleotides were purchased from Eurofins Genomics (Ebersberg, Germany) on a 0.2 μmol synthesis scale with reverse phase high performance liquid chromatography (RP-HPLC) purification and subsequent quality control by mass spectrometry.

The primer sequences used for the routine method were obtained from Brandal *et al*., 2007 and are listed in Table 1 [7]. The nine sets of Cliffhanger primers listed in Table 2 were designed to amplify five categories of DEC, with the *rrs* gene from *E*. *coli* as an internal control [13]. The Cliffhanger primers were designed to meet four criteria: 1) the primers were designed to cover all subtypes of each gene, 2) the target annealing temperature of each primer was 60°C, 3) the amplicon lengths were between 100 and 180 base pairs and 4) the target GC content of each primer was 40% to 60%. In all single-stranded custom-designed DNA overhangs (X) a single 3’ adenine nucleobase was introduced 5’ to the internal *ortho*-TINA monomer (Z) to space between the *ortho*-TINA monomer in the Cliffhanger primer and the complementary DNA Taq sequence on the MagPlex microspheres (Fig 1). Each Cliffhanger primer pair was tested on boiled colony material with SYBR green I detection to ensure PCR efficiency above 98% in a singleplex reaction. The Cliffhanger primers for all nine genes were subsequently mixed and tested using multiplex PCR with positive controls. The primer concentrations were adjusted to avoid off-target amplification, to obtain comparable MFIs for all targets on the MagPix instrument and to ensure that the internal control (*rrs*) had a lower MFI than all other targets.

**Table 1:** Routine multiplex primers as reported by Brandal et al, 2007.

Primer name	DNA sequence	Primer concen-tration (nM)	Amplicon length (bp)
*rrs* F	CCCCCTGGACGAAGACTGAC	200	401
*rrs* R	ACCGCTGGCAACAAAGGATA	200	
*eae* F	TCAATGCAGTTCCGTTATCAGTT	200	482
*eae* R	GTAAAGTCCGTTACCCCAACCTG	200	
*stx1* F	AAATCGCCATTCGTTGACTACTTCT	200	370
*stx1* R	TGCCATTCTGGCAACTCGCGATGCA	200	
*stx2* F	CAGTCGTCACTCACTGGTTTCATCA	200	283
*stx2* R	GGATATTCTCCCCACTCTGACACC	200	
*ipaH* F	GTTCCTTGACCGCCTTTCCGATACCGTC	200	619
*ipaH* R	GCCGGTCAGCCACCCTCTGAGAGTAC	200	
*aggR* F	GTATACACAAAAGAAGGAAGC	200	254
*aggR* R	ACAGAATCGTCAGCATCAGC	200	
*elt* F	TCTCTATGTGCATACGGAGC	200	322
*elt* R	CCATACTGATTGCCGCAAT	200	
*estAh* F	ATTTTTCTTTCTGTATTGTCTT	200	190
*estAh* R	CACCCGGTACAAGCAGGATT	200	

**Table 2:** Cliffhanger multiplex primers consisted of a 5’-positioned *ortho-*TINA molecule (Z), a custom-designed ssDNA overhang, an internally placed *ortho*-TINA molecule (Z) and a target-specific primer sequence. Reverse primers were labelled with af 5’-positioned biotin (Bio) and an *ortho-*TINA molecule (Z).

Primer name	Oligonucleotide sequence	Primer concen-tration (nM)	Amplicon length (bp)
*rrs* F	ZGTCCGCAGCCAACCAAACGC-AZAGGCAGCAGTGGGGAATA	25	176
*rrs* R	Bio-ZGTGCTTCTTCTGCGGGTAA	25	
*eae* F	ZCACCGCAGCCTCCCAACCAA-AZATCAGGATTTTTCTGGTGATAATACCC	100	162
*eae* R	Bio-ZGGCGCTCITCATAGTCTTTCTT	100	
*stx1* F	ZTGGCGGAACAGGACTGCGGA-AZACAGGACAAAIAATGTTTTTTATCGCTTT	100	181
*stx1* R	Bio-ZGTCAICGAATGGCGATTTATCTGCA	100	
*stx2* F	ZGACGCCAACGGACGGAGGGT-AZTCCATGACIACGGACAGCAGITAT	200	137
*stx2* R	Bio-ZAACTCCATTAAIICCAGATATGATGAA	200	
*ipaH* F	ZCGAGGGAAGTGGGCAGCGGA-AZGATTCCGTGAACAGGTCGCTG	25	156
*ipaH* R	Bio-ZGGAGATTGTTCCATGTGAGCG	25	
*aggR* F	ZGGGTGGAAAGCGGAGCGTGG-AZCACAAAAGAAGGAAGCAATACA	175	127
*aggR* R	Bio-ZTGGATTTACTGTTGATTTCTTCT	175	
*elt* F	ZGCGAGGGTGCGAGGGTTGCT-AZGGATGGTATCGTGTTAATTTTGG	100	148
*elt* R	Bio-ZGAAACCTGCTAATCTGTAACCA	100	
*estAh* F	ZGCGAGCGAACCAGAGCGACG-AZTTTCICTCAGGATGCTAAACCA	100	166
*estAh* R	Bio-ZATTACAACACAATTCACAGCAGTAA	100	
*estAp* F	ZGTCCTGCTGTGGGCGATGGC-AZTTGACTCTTCAAAAGAGAAAATTACATTAGA	100	154
*estAp* R	Bio-ZGCACAGGCAGGATTACAACAAAG	100	

### Multiplex PCR

The routine multiplex PCR assay was performed in a 25-μL reaction volume using 1x QIAGEN Multiplex PCR Master Mix, 1x Primer Mix (Table 1), 1x Q-solution and 1 μL of the lysed specimen in a capped 0.2 mL Eppendorf tube. All PCR assay tubes were set up manually on ice. PCR was performed on Veriti Thermal Cycler (Applied BioSystems, Nærum, Denmark) utilizing the following cycling conditions: Hot-Start polymerase activation for 15 minutes at 95°C; 30 cycles of denaturation at 95°C for 30 seconds; annealing at 57°C for 90 seconds; and elongation at 72°C for 90 seconds with a final elongation step at 72°C for 10 minutes after the last PCR cycle.

Cliffhanger PCR was performed in a 25 μL reaction volume using in-house DEC buffer (10.4 mM Tris-HCl, 56.8 mM Trizma-base, 16.1 mM (NH_4_)_2_SO_4_, 0.01% Tween 80), 0.03% Triton X-100, 3 mM MgCl_2_, 0.08% Bovine Serum Albumin (non-acetylated), 0.2 mM of each dNTP (a mixture of 0.066 mM dTTP and 0.133 mM dUTP was used), 0.625 units of Uracil N-Glycosylase (UNG, cat. no. EN0361, Fermentas GmbH, St. Leon-Rot, Germany), 1 unit of KAPA2G Robust HS (KapaBiosystems, Cape Town, South Africa), 1x Primer Mix (Table 2) and 5 μL of the target lysate in an ABgene^®^ SuperPlate^™^ 96-well PCR plate (ABgene, Epsom, United Kingdom) sealed with optically clear, adhesive Microseal^®^ "B" Film (BioRad Laboratories, Copenhagen, Denmark). All PCRs were set up manually on ice. PCR was performed using the CFX96^™^ Real-Time System (BioRad Laboratories, Copenhagen, Denmark) and the following cycling conditions: UNG treatment for 10 minutes at 40°C; UNG inactivation and Hot-Start polymerase activation for 10 minutes at 95°C; 35 cycles of denaturation at 95°C for 15 seconds; annealing at 64°C for 30 seconds; and elongation at 72°C for 30 seconds.

### Detection of multiplex PCR products

For the routine method, PCR amplicons were detected on the Qiaxcel System (Qiagen, Copenhagen, Denmark). The plates, with 25 μL of PCR amplicons, were loaded into the Qiaxcel System and analysed using the QIAxcel High Resolution Cartridge.

For the Cliffhanger method, 5 μL of PCR product was mixed with 0.2 μL/well of each MagPlex microsphere that was coated according to Luminex recommendations with a complementary ZIP-sequence for each PCR amplicon in a 96-well polystyrene conical bottom MicroWell plate (Nunc, Thermo Fisher Scientific, Roskilde, Denmark) (S2 Table). The incubation volume was adjusted to 100 μL by adding hybridization buffer for a final concentration of 20 mM NaH_2_PO_4_/Na_2_HPO_4_, pH 7.2, 400 mM NaCl and 0.03% Triton X-100. The plate was sealed with adhesive aluminum foil, and the mixture was incubated at 54°C for 30 minutes at 900 rpm in an iEMS Incubator/Shaker HT (Thermo Fisher Scientific, Nærum, Denmark). Subsequently, the plate was washed three times in 100 μL of wash buffer (5 mM NaH_2_PO_4_/Na_2_HPO_4_, pH 7.2, 100 mM NaCl and 0.03% Triton X-100). Each wash cycle consisted of 1 minute of sedimentation on a 96-well magnetic separator (PerkinElmer, Skovlunde, Denmark), removal of the buffer with an 8-channel pipette at speed 1 (eLINE, Biohit multichannel electronic pipette), the addition of 100 μL of wash buffer and incubation at room temperature for 1 minute. After the washing steps, 100 μL of hybridization buffer spiked with a final concentration of 5 μg/mL of Streptavidin-R-PhycoErythrin Premium Grade (S-21388, Invitrogen A/S, Tåstrup, Denmark), then 100 μg/mL of albumin fraction V (Merck Millipore, Hellerup, Denmark) was added and the mixture was incubated at 54°C for 15 minutes at 900 rpm in an iEMS Incubator/Shaker HT. Three additional washing steps were performed as described above, and the volume was adjusted to 100 μL/well. The microtiter plate was transferred to the MagPix instrument (Luminex, Austin, TX, USA), and 150 μL of fluid per well was analysed, counting a minimum of 100 beads per analyte and with the plate temperature set to 47°C. Each plate had the three positive controls plus five wells containing negative controls, and each clinical sample was run in a single well. Clinical samples were considered positive if the MFI was above the mean MFI of the five negative controls plus three standard deviations. All samples that were negative for the *rrs* gene were re-run after 8-fold and 20-fold dilutions to ensure that inhibition was not causing the internal control to appear negative.

### Denaturation assays

The sensitivity of the Cliffhanger method was compared to probe based detection of denatured dsDNA PCR amplicons for the *eae*, *aggR* and *ipaH* targets. PCR amplicons were generated using the Cliffhanger multiplex PCR assay described above using unmodified DNA primers and *ortho*-TINA modified primers to amplify the targets from the first (*aggR*) and third (*eae* and *ipaH*) positive controls. The sensitivity of the two methods was compared using a two-fold dilution series of biotinylated PCR products equal to 59524 CFU/well to 58 CFU/well. The Cliffhanger assays were conducted as described above. The dsDNA PCR products were denatured and competitively reannealed to MagPlex microspheres, and the assays were performed as described previously [14]. In brief, 70 μL of a premix of MagPlex microspheres, PCR product and hybridization buffer was mixed in an Eppendorf twin.tec 96-well PCR plate and incubated at 95°C for 10 minutes in a SensoQuest Labcycler (SensoQuest GmbH, Göttingen, Germany). The PCR plate was then immediately placed on ice for 2 minutes, and 50 μL of the premix was transferred to a 96-well polystyrene conical bottom MicroWell plate (Nunc, Thermo Fisher Scientific, Roskilde, Denmark). Each well had a mixture of 0.2 μL/well of MagPlex microspheres to detect the *eae*, *ipaH* and *aggR* targets (S2 Table), a two-fold dilution series of biotinylated PCR product from 59524 to 58 CFU/well and hybridization buffer that had a final concentration of 20 mM NaH_2_PO_4_/Na_2_HPO_4_, pH 7.2, 400 mM NaCl and 0.03% Triton X-100. The plate was sealed, incubated, washed, detected with Streptavidin-R-PhycoErythrin and run on the MagPix instrument following the same procedure as for the Cliffhanger assay.

### Sanger sequencing

Sanger sequencing of samples that were positive by the Cliffhanger method and negative by the routine method was performed using a slightly modified version of the multiplex Cliffhanger PCR assay as described above. The amplified targets were amplified further using target-specific primers without the Cliffhanger overhang for each target (Table 2). For each singleplex PCR, 200 nM of the forward and reverse primers were used for the amplification along with 5 μL of the diluted PCR amplicon as the target. Each PCR amplicon was diluted 100-fold before use. The amplification of each target was checked by gel electrophoresis on a 1.5% agarose gel in TAE buffer with ethidium bromide staining and the GeneRuler 100 bp Plus DNA Ladder (Fermentas GmbH, St. Leon-Rot, Germany). These PCR products were sent to Macrogen Europe (Amsterdam, the Netherlands) for purification and Sanger sequencing. The sequencing reactions were performed using the primer sequence portion of each Cliffhanger primer (Table 2, unmodified DNA primers). The sequencing data were annotated by nucleotide BLAST on the NCBI home page. An annotation was considered valid if a target unique oligonucleotide sequence was identified (excluding the primer sequences).

### Statistical analysis

The two-by-two table for each gene is reported in S3 Table and was evaluated by the chi-squared test. The level of significance was set to 95%. Clinical sensitivity, specificity, PPV and NPV value were calculated considering all positive samples as true positives as they either were culture positive or verified by Sanger sequencing.

## Results

### Validation of the analytical sensitivity of the Cliffhanger method versus detection of denatured PCR amplicons

**Fig 3:**
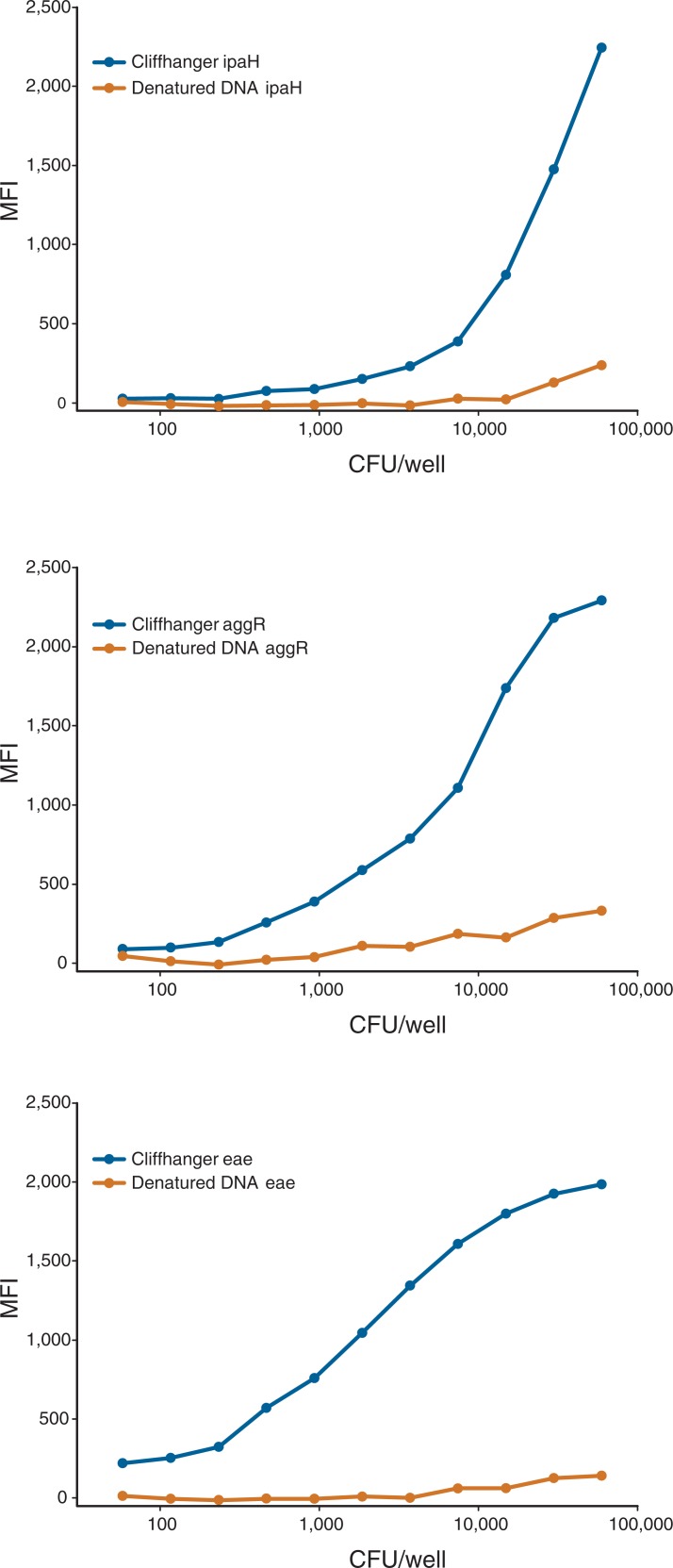
The analytical sensitivity of Cliffhanger overhangs versus denatured dsDNA. The median fluorescence intensity (MFI) is reported as the MFI after subtraction of the median background MFI. The limit of detection of the Cliffhanger method was equal to an MFI of 59 for *eae*, 83 for *aggR* and 68 for *ipaH*. The limits of detection for the denatured PCR amplicons were equal to an MFI of 64 for *eae*, 71 for *aggR* and 68 for *ipaH*.

We first compared the analytical sensitivity of the Cliffhanger method versus the sensitivity of PCR amplicons that were denatured prior to hybridization with the specific probe. The primer sequences were identical for the two methods, but the Cliffhanger primers contained an additional *ortho*-TINA molecule at the 5´-position plus a capture sequence positioned 5´ to the *ortho*-TINA molecule (Fig 1). The limit of detection was set as the mean background MFI plus three standard deviations. The Cliffhanger method detected the *eae* and *aggR* genes in the most diluted PCR reactions (the equivalent of 58 colony-forming units, CFUs, of *E*. *coli*) and detected the *ipaH* gene at the equivalent of 465 CFU. In contrast, using unmodified DNA primers followed by detection by denaturation of the PCR amplicon on the MagPix instrument, the *ipaH* gene could only be detected using 29,762 CFU of *E*. *coli*. For the *eae* gene, denatured PCR amplicons needed 7440 CFU of *E*. *coli* for detection, and for the *aggR* gene, it needed the equivalent of 1860 CFU of *E*. *coli*. Thus, the Cliffhanger method was 32- to 128-fold more sensitive than denaturation of the PCR amplicon when lysed bacterial colonies were used as templates for the PCR assays. The MFI values after subtraction of the median background MFI are shown in Fig 3. For all three target genes, i.e. *eae*, *aggR* and *ipaH*, the Cliffhanger method detected significantly higher MFI signals. As the reported MFI and analytical sensitivity was much higher with the Cliffhanger method, only the Cliffhanger method was used subsequently for the clinical evaluation in which we compared it with the routine method for DEC detection.

### Method validation using a control DEC strain collection

To ensure that the Cliffhanger method would detect DEC isolated from clinical samples, we tested a collection of 105 *E*. *coli* strains that were provided by the Statens Serum Institute (SSI), Copenhagen, Denmark. The results are summarised in Table 3 and in S1 Table. The strain collection was tested using both the routine method and the Cliffhanger method. Both the routine method and the Cliffhanger method missed one *stx1*-positive sample but correctly verified 16 *stx1*-positive samples and 88 *stx1*-negative samples. The sensitivity for *stx1* was 94.1% using both the routine method and the Cliffhanger method, and the specificity was 100% for both methods. Both the routine method and the Cliffhanger missed one *stx2-*positive sample but correctly verified 19 *stx2*-positive samples and 85 *stx2-*negative samples. The sensitivity for *stx2* was 95% using both the routine method and the Cliffhanger method, and the specificity was 100% for both methods. The Cliffhanger method missed three *estAp*-positive samples, two of which were expected to be positive for both the *estAh* and *estAp* genes; however, only the *estAh* gene was detected by the Cliffhanger method. The Cliffhanger method verified five *estAp*-positive samples, but these samples could not be evaluated using the routine method, as *estAp* is not included in the multiplex PCR that we used to detect the genes by the routine method. The Cliffhanger method identified 97 *estAp*-negative samples. The sensitivity for *estAp* was 62.5% using the Cliffhanger method, and the specificity was 100%. Both the routine method and the Cliffhanger correctly verified the following: two *aggR-*positive and 103 *aggR*-negative samples; eight *estAh-*positive samples and 97 *estAh*-negative samples; 13 *ipaH-*positive and 92 *ipaH-*negative samples; 15 *elt*-positive and 90 *elt-*negative samples; and 63 *eae-*positive and 42 *eae-*negative samples. The sensitivity and specificity for *aggR*, *estAh*, *ipaH*, *elt*, *eae* and *rrs* was 100% using both the routine method and the Cliffhanger method. These results verified that the performance of the Cliffhanger method was satisfactory compared to the routine method using material from directly lysed bacterial colonies. Accordingly, we next performed a clinical study in which the Cliffhanger method was used to directly analyse lysed faecal samples.

**Table 3:** Testing of 105 DEC strains from a reference collection by the routine method and by the Cliffhanger method.

105 *E*. *coli* strains	Number of strains that were positive for each gene								
	*estAp*	*estAh*	*elt*	*eae*	*stx1*	*stx2*	*aggR*	*ipaH*	*rrs*
**Reference collection**	**8**	**8**	**15**	**63**	**17**	**20**	**2**	**13**	**105**
**Routine method**	**-**	**8**	**15**	**63**	**16**	**19**	**2**	**13**	**105**
**Cliffhanger method**	**5**	**8**	**15**	**63**	**16**	**19**	**2**	**13**	**105**

### Comparing the clinical sensitivity of the routine method to the Cliffhanger method

**Fig 4:**
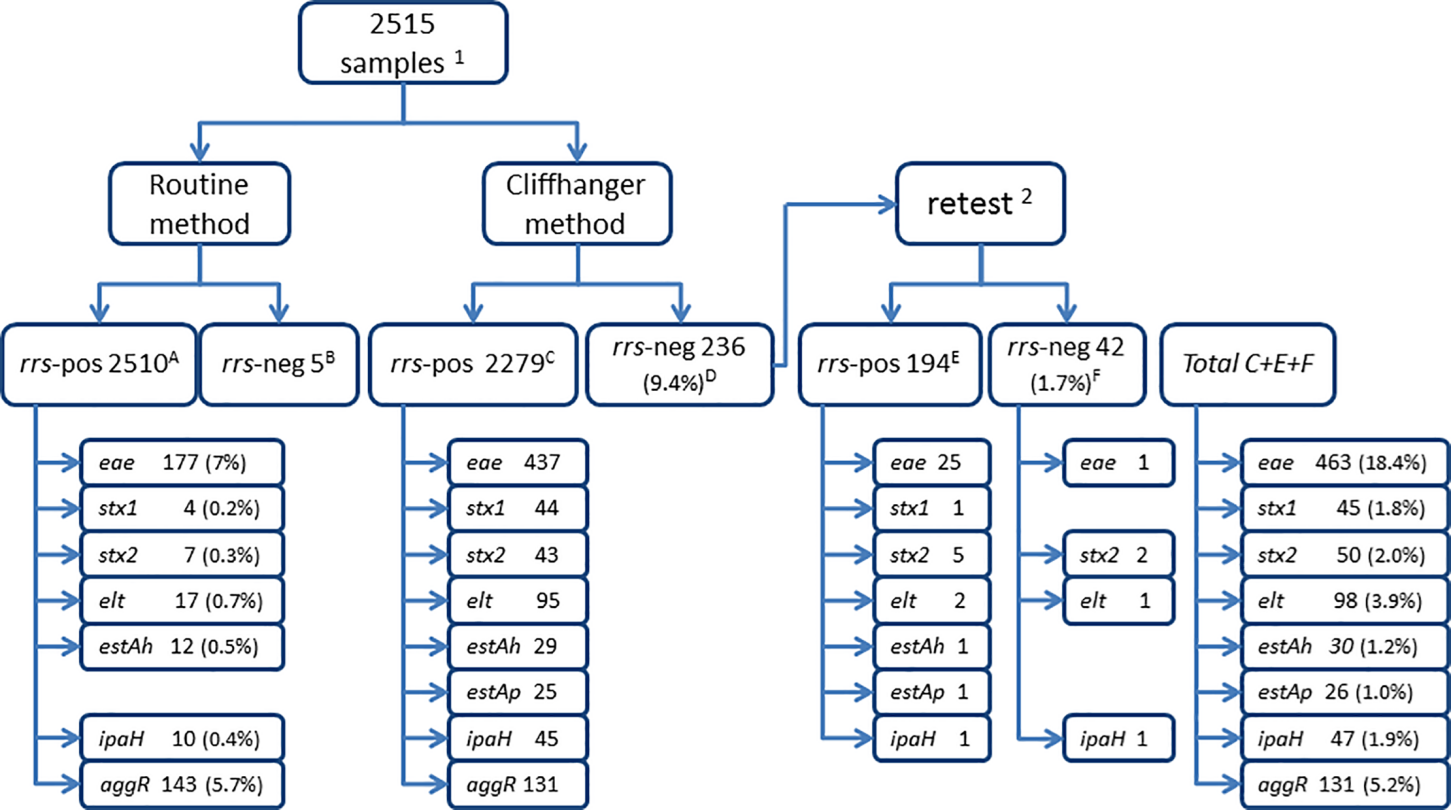
Positive findings using the routine method versus the Cliffhanger method. ^1^Samples were collected from Dec 1, 2012 to March 20, 2013. ^2^For retesting, the samples were diluted 8-fold and 20-fold prior to multiplex PCR. Pos, positive; neg, negative. The number of positives and the percentages of all samples that were positive are shown for each gene.

**Fig 5:**
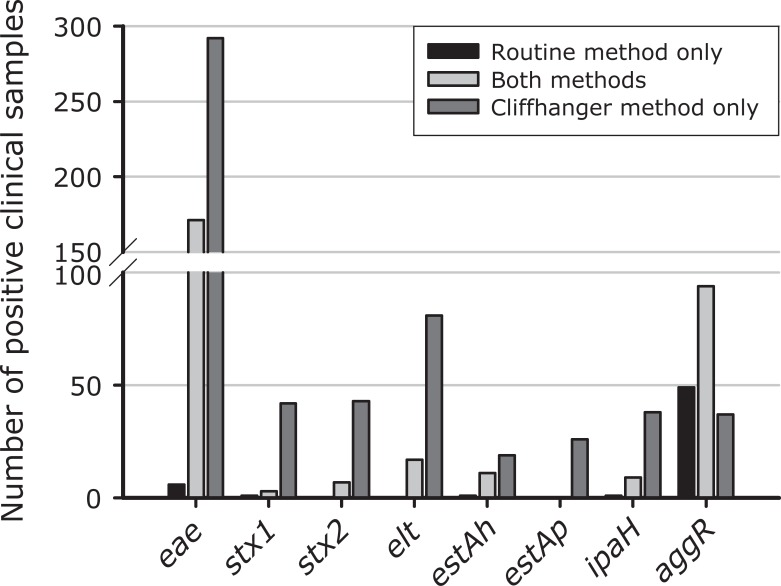
Detecting DEC pathogens in clinical samples. The samples are reported as being either positive by both methods or positive by only one of the methods. All samples that were positive only by the Cliffhanger method were verified by Sanger sequencing.

We used both the routine method and the Cliffhanger method to analyse 2515 clinical faecal samples from patients with diarrhoea. The positive rates were significantly higher using the Cliffhanger method for all genes except the *aggR* gene. Fig 4 shows the number of positive samples for each method, and Fig 5 and S3 Table show the agreement between the two methods for each gene. For enteropathogenic *E*. *coli*, the routine method identified 177 *eae*-positive samples (7.0%), while the Cliffhanger method identified 463 *eae*-positive samples (18.4%) (P<0.0001). The sensitivity for *eae* was 37.7% and the negative predictive value (NPV) was 87.5% by the routine method, and the sensitivity for *eae* was 98.7% and NPV was 99.7% by the Cliffhanger method. For Shiga toxin-producing *E*. *coli*, the routine method identified four *stx1-*positive samples (0.2%), while the Cliffhanger method identified 45 *stx1-*positive samples (1.8%) (P<0.0001); with a sensitivity of 8.7% and NPV of 98.3% by the routine method and a sensitivity of 97.8% and NPV of 99.96% by the Cliffhanger method. The routine method identified seven *stx2-*positive samples (0.3%), while the Cliffhanger method identified 50 *stx2-*positive samples (2.0%) (P<0.0001); with a sensitivity of 14.0% and NPV of 98.3% by the routine method and a sensitivity and NPV of 100% by the Cliffhanger method. For enteroinvasive *E*. *coli*, the routine method identified ten *ipaH*-positive samples (0.4%), while the Cliffhanger method identified 47 *ipaH*-positive samples (1.9%) (P<0.0001); with a sensitivity of 20.8% and NPV of 98.5% by the routine method and a sensitivity of 97.9% and NPV of 99.96% by the Cliffhanger method. For enterotoxigenic *E*. *coli*, the routine method could not be used to test for *estAp*, but the Cliffhanger method identified 26 *estAp*-positive samples (1.0%). For the *elt* gene, the routine method identified 17 *elt*-positive samples (0.7%), while the Cliffhanger method identified 98 *elt*-positive samples (3.9%) (P<0.0001); with a sensitivity of 17.3% and NPV of 96.8% by the routine method and a sensitivity and NPV of 100% by the Cliffhanger method. For the *estAh* gene, the routine method identified 12 *estA-*positive samples (0.5%), while the Cliffhanger method identified 30 *estA-*positive samples (1.2%) (P<0.0001); with a sensitivity of 38.7% and NPV of 99.2% by the routine method and a sensitivity of 96.8% and NPV of 99.96% by the Cliffhanger method. Interestingly, for enteroaggregative *E*. coli, the routine method identified 143 *aggR*-positive samples (5.7%), which was more than for the Cliffhanger method, which identified 131 *aggR*-positive samples (5.2%) (P = 0.197); with a sensitivity of 79.4% and NPV of 98.4% by the routine method and a sensitivity of 72.8% and NPV of 97.9% by the Cliffhanger method. The specificity and positive predictive value (PPV) was 100% for all targets by both methods.

As shown in Fig 5, none of the samples were *stx2*-positive or *elt*-positive only by the routine method, whereas 43 samples were *stx2*-positive and 81 samples were *elt*-positive only by the Cliffhanger method. One sample was *stx1*-positive, one was *estAh*-positive and one was *ipaH-*positive only by the routine method, whereas 42 samples were *stx1-*positive, 19 samples were *estAh-*positive and 38 samples were *ipaH-*positive only by the Cliffhanger method. For *eae*, six samples were positive only by the routine method, while 292 were positive only by the Cliffhanger method. Finally, 49 samples were *aggR*-positive only by the routine method, while 37 samples were *aggR*-positive only by the Cliffhanger method.

Using the Cliffhanger method, 236 samples (9.4% of all samples) were negative for the positive control (the *rrs* gene) on the first run (Fig 4). These samples were re-evaluated at both eight-fold and 20-fold dilutions (relative to the initial samples) to overcome potential sample inhibition. After the rerun, 42 samples (1.7% of all samples) were still negative for the *rrs* gene by the Cliffhanger method. Five of these 42 samples were found to be positive for DEC genes by the Cliffhanger method (one *elt*-positive, one *eae*-positive, two *stx2*-positive and one *ipaH*-positive). One of the 42 samples that was *rrs-*negative by the Cliffhanger method was *aggR*-positive by the routine method. Five samples were *rrs*-negative by the routine method; all of these five samples were *rrs* positive including one *eae*-positive and one *elt*-positive sample by the Cliffhanger method. None of these five samples were positive for DEC genes by the routine method.

The PCR products for all samples that were positive for DEC genes only by the Cliffhanger method were sent for Sanger sequencing to ensure that this method specifically amplified DEC genes. A total of 578 PCR products that were only positive by the Cliffhanger method were verified by Sanger sequencing: 292 *eae*-positive samples, 42 *stx1*-positive samples, 43 *stx2*-positive samples, 81 *elt*-positive samples, 19 *estAh*-positive samples, 26 *estAp*-positive samples, 38 *ipaH*-positive samples and 37 *aggR*-positive samples (Fig 5 and S3 Table).

### A comparison of the findings in the clinical samples between the two methods

A total of 706 of the 2515 samples (28.1%) were positive for one or more DEC genes by one of the methods (Table 4); 220 samples (8.7%) were positive for an equal number of DEC genes by both methods; 44 samples (1.7%) were positive for more DEC genes by the routine method; and 442 samples (17.6%) were positive for more toxin genes by the Cliffhanger method. Of the 2176 samples that were negative for DEC genes by the routine method, 23 were positive for three or more DEC genes by the Cliffhanger method. One sample was only positive by the Cliffhanger method for six genes, including STEC (*stx1*, *stx2*), ETEC (*estAh*, *elt*), EIEC/shigella (*ipaH*) and possible EPEC (*eae*). Five samples were positive only by the Cliffhanger method for four genes: one sample was positive for STEC (*stx1*, *stx2*), ETEC (*elt*) and possible EPEC (*eae*); three samples were positive for STEC (*stx1*), ETEC (*estAh*, *elt*) and possible EPEC (*eae)*; and one sample was positive for ETEC (*estAh*, *estAp*, *elt*) and possible EPEC (*eae*). Two samples were positive only by the routine method for two genes, namely possible EPEC (*eae)* and EAEC (*aggR)*.

**Table 4:** Number of DEC genes in clinical samples that were positive by the routine method versus the Cliffhanger method.

		Cliffhanger method			
		0	1	2	≥3
**Routine method**	**0**	**1809**	**301**	**43**	**23**
	**1**	**33**	**211**	**45**	**20**
	**2**	**2**	**9**	**8**	**10**
	**≥3**	**0**	**0**	**0**	**1**

## Discussion

Since this study ended in 2013, a number of commercial multiplex PCR assays for gastrointestinal infections have become available. Several of these assays are well-suited for use in small laboratories, as they test one sample at a time and are relatively expensive to run [9,10]. Large laboratories may utilise commercially available assays as well; however, due to the cost and laboratory workflow, larger labs sometimes instead use laboratory-developed tests (LDTs) if they are permitted by local regulations [15,16]. The major materials costs for LDTs are plastic goods and nucleic acid extraction reagents. In this study, we used a simple lysis protocol together with Cliffhanger primers, which allowed us to conduct multiplex PCR directly on the majority of crude lysates.

The internal *rrs* control was not amplified by the Cliffhanger method for 236 of the 2515 samples in the initial run. These samples were re-run after diluting the samples 8-fold and 20-fold; after the re-run, there were 42 samples in which the *rrs* gene was not amplified. DEC genes were identified in five of these 42 samples at the re-run, leaving 37 samples (1.5%, seven from dry containers and 30 from FecalSwab media) that were completely negative for DEC genes by the Cliffhanger method. The samples that were negative for the *rrs* gene using the Cliffhanger method appeared to be denser than other samples; accordingly, they may therefore simply have more PCR inhibitors compared to other stool samples or, alternatively, the samples may have been from patients with formed stool. To reduce the frequency of false negative samples, it may be useful to use rectal swabs instead of stool samples for the diagnosis of DEC, as formed stool may interfere with nucleic acid preparation procedures and with the subsequent multiplex PCR [17]. We did not dilute the samples more than 20-fold because this could increase the risk of the samples being falsely negative for the DEC genes. All 42 samples that were *rrs*-negative by the Cliffhanger method were found to contain *E*. *coli* by the routine method, suggesting that all 2515 samples tested contained *E*. *coli*.

All of the clinical samples that were included in this study came from patients who had diarrhoea according to the ordering physician. Use of the Cliffhanger method increased the gene positivity rate for DEC specimens to 26.7% compared to 13.5% by the routine method. This was expected, as the routine method uses a smaller culture volume of the clinical specimens. Specifically, the routine method analyses selected colonies from 5 μL of starting material, whereas the Cliffhanger method analyses all of the DNA that is present in 200 μL of sample; this significantly increases the positivity rate for potentially clinically relevant DEC genes [16]. The majority of the clinical samples included in this study (2005 of the 2515 samples) were collected by rectal swab at the patient’s bedside. Although this is convenient for both healthcare professionals and for the patient, the sensitivity may be lower compared to direct sampling of faeces since less material is collected [16].

In addition to checking that the DEC genes are truly present in the clinical samples when developing an LDT, it is still necessary to culture the samples that are positive for *eae*, *ipaH*, *stx1* and *stx2* to identify clinically relevant DEC isolates [5]. Currently, molecular methods should not stand alone for evaluating DEC-positive samples. However, due to their increased sensitivity compared to culture-based methods, they may be used to rule out DEC as a cause of diarrhoea when they are negative and thereby prevent repeated sampling of patients with diarrhoea [16]. Likewise, commercial multiplex PCR assays for pathogenic bacteria in human faeces can detect the bacteria at the genus level, but they do not discriminate between pathogenic and non-pathogenic species. Additional testing is thus needed to determine the clinical relevance of a positive test. Assessment by metagenome sequencing could represent an alternative method. However, the technology is still limited by sample throughput, by longer total turn-around time, by price, by the choice of nucleic acid purification method and by the lack of close matches for annotation, especially for viruses [18,19].

In the present study, we found that the Cliffhanger multiplex PCR method was more sensitive than the routine method except for detecting *aggR*-positive samples. EAEC strains can be pathogenic or non-pathogenic, and it may be necessary to detect several genes to determine whether an EAEC strain is associated with diarrhoea [20]. The *aggR* gene is one of several relevant genes, but the current Cliffhanger primers for *aggR* need to be redesigned as these primers missed an important number of *aggR*-positive samples compared to the culture-based routine method.

Both the routine method and the Cliffhanger method were evaluated using a collection of 105 clinical reference isolates from SSI. Both methods missed a *stx1*- and a *stx2*-positive sample. It is interesting that both methods missed the same two reference isolates, since different primers were used for the two PCR analyses. This raises the question of whether these two reference samples are truly positive for the *stx1* and *stx2* genes. Three *estAp*-positive samples in the clinical reference isolate collection were missed by the Cliffhanger method. The *estAp*-positive samples in the clinical reference isolate collection were verified by PCR but not by sequencing by the SSI (The International Collaborating Centre for Reference and Research on Escherichia and Klebsiella, personal communication). The estAp primers that SSI uses to classify the clinical reference sample collection are not specific for *estAp* since they can cross-react with *estAh (*The International Collaborating Centre for Reference and Research on Escherichia and Klebsiella, personal communication). Two of the reference samples that should have been positive for both *estAh* and *estAp* may have been misclassified by SSI as being positive for both *estAh* and *estAp*. There is no clear explanation for why for the last *estAp*-positive reference sample was missed by the Cliffhanger method. It may be that the Cliffhanger primers were so specific that they did not cross-react with other genes in the clinical isolate collection or an *E*. *coli* isolate that did not carry the *estAp* gene by accident was frozen and included in the reference collection, as the reference collection has been passaged multiple times since it was established for commercial use.

The additional findings by the Cliffhanger method were not used to change patient management, for example to avoid antibiotic therapy for STEC or to start antibiotic treatment for *Shigella spp*. or EPEC after additional phenotypic identification. Indeed, the potential clinical impact on patient management of commercially available multiplex molecular testing for gastrointestinal pathogens has not been thoroughly evaluated. These tests demonstrate enhanced detection of pathogens, but they cost at least twice more than LDT [21]. Using an LDT could improve pathogen detection and reduce total sample turn-around time at a lower cost. Indeed, direct testing of lysed faecal material along with the limited number of purification steps used in the Cliffhanger method may decrease sample turn-around time and the cost of testing clinical samples for gastrointestinal pathogens. However, the assay must be developed further to include additional relevant clinical gastrointestinal pathogens. Cliffhanger primers could potentially be used to develop any multiplex PCR-based assay that relies on oligonucleotide probe hybridisation.

## Supporting information

S1 Table Analysis of the Diarrhoeagenic *Escherichia coli* control strain collection by the routine method versus the Cliffhanger method. (DOC)

S2 Table The Zip-sequences and MagPlex magnetic microspheres used in this study. (DOC)

S3 Table Two-by-two tables for the target genes shown in Fig 4. (DOC)

## References

[1] Wang M, Szucs TD, Steffen R. Economic aspects of travelers' diarrhea. J Travel Med. 2008; 15: 110–118. doi: 10.1111/j.1708-8305.2008.00189.x 18346244 10.1111/j.1708-8305.2008.00189.x

[2] Kaper JB, Nataro JP, Mobley HL. Pathogenic *Escherichia coli*. Nat Rev Microbiol. 2004; 2: 123–140. doi: 10.1038/nrmicro818 15040260 10.1038/nrmicro818

[3] Persson S, Olsen KE, Scheutz F, Krogfelt KA, Gerner-Smidt P. A method for fast and simple detection of major diarrhoeagenic *Escherichia coli* in the routine diagnostic laboratory. Clin Microbiol Infect. 2007; 13: 516–524. doi: 10.1111/j.1469-0691.2007.01692.x 17331124 10.1111/j.1469-0691.2007.01692.x

[4] Delannoy S, Beutin L, Fach P. Discrimination of Enterohemorrhagic *Escherichia coli* (EHEC) from non-EHEC strains based on detection of various combinations of type III effector genes. J Clin Microbiol. 2013; 51: 3257–3262. doi: 10.1128/JCM.01471-13 23884997 10.1128/JCM.01471-13 PMC3811616

[5] Nataro JP, Kaper JB. Diarrheagenic *Escherichia coli*. Clin Microbiol Rev. 1998; 11: 142–201. 9457432 10.1128/cmr.11.1.142 PMC121379

[6] Bonkoungou IJ, Lienemann T, Martikainen O, Dembelé R, Sanou I, Traoré AS, et al Diarrhoeagenic *Escherichia coli* detected by 16-plex PCR in children with and without diarrhoea in Burkina Faso. Clin Microbiol Infect. 2012; 18: 901–906. doi: 10.1111/j.1469-0691.2011.03675.x 21985619 10.1111/j.1469-0691.2011.03675.x

[7] Brandal LT, Lindstedt BA, Aas L, Stavnes TL, Lassen J, Kapperud G. Octaplex PCR and fluorescence-based capillary electrophoresis for identification of human diarrheagenic *Escherichia coli* and *Shigella* spp. J Microbiol Methods 2007; 68: 331–341. doi: 10.1016/j.mimet.2006.09.013 17079041 10.1016/j.mimet.2006.09.013

[8] Ngwa GA, Schop R, Weir S, León-Velarde CG, Odumeru JA. Detection and enumeration of *E*. *coli* O157:H7 in water samples by culture and molecular methods. J Microbiol Methods 2013; 92: 164–172. doi: 10.1016/j.mimet.2012.11.018 23220187 10.1016/j.mimet.2012.11.018

[9] Binnicker MJ. Multiplex molecular panels for diagnosis of gastrointestinal infection: Performance, result interpretation, and cost-effectiveness. J Clin Microbiol. 2015; 53: 3723–3728. doi: 10.1128/JCM.02103-15 26311866 10.1128/JCM.02103-15 PMC4652086

[10] Buss SN, Leber A, Chapin K, Fey PD, Bankowski MJ, Jones MK, et al Multicenter evaluation of the BioFire FilmArray gastrointestinal panel for etiologic diagnosis of infectious gastroenteritis. J Clin Microbiol. 2015; 53: 915–925. doi: 10.1128/JCM.02674-14 25588652 10.1128/JCM.02674-14 PMC4390666

[11] Wilson IG. Inhibition and facilitation of nucleic acid amplification. Appl Environ Microbiol. 1997; 63: 3741–3751. 9327537 10.1128/aem.63.10.3741-3751.1997 PMC168683

[12] Schneider UV, Mikkelsen ND, Lindqvist A, Okkels LM, Jøhnk N, Lisby G. Improved efficiency and robustness in qPCR and multiplex end-point PCR by twisted intercalating nucleic acid modified primers. PLoS One. 2012; 7: e38451 doi: 10.1371/journal.pone.0038451 22701644 10.1371/journal.pone.0038451 PMC3368873

[13] Wilson KH, Blitchington RB, Greene RC. Amplification of bacterial 16S ribosomal DNA with polymerase chain reaction. J Clin Microbiol. 1990; 28: 1942–1946. 2095137 10.1128/jcm.28.9.1942-1946.1990 PMC268083

[14] Schneider UV, Géci I, Jøhnk N, Mikkelsen ND, Pedersen EB, Lisby G. Increasing the analytical sensitivity by oligonucleotides modified with *para*- and *ortho*-twisted intercalating nucleic acids—TINA. PLoS. One. 2011; 6: e20565 doi: 10.1371/journal.pone.0020565 21673988 10.1371/journal.pone.0020565 PMC3108614

[15] Perry MD, Corden SA, Howe RA. Evaluation of the Luminex xTAG gastrointestinal pathogen panel and the Savyon Diagnostics gastrointestinal infection panel for the detection of enteric pathogens in clinical samples. J Med Microbiol. 2014; 63: 1419–1426. doi: 10.1099/jmm.0.074773-0 25102908 10.1099/jmm.0.074773-0

[16] Liu J, Kabir F, Manneh J, Lertsethtakarn P, Begum S, Gratz J, et al Development and assessment of molecular diagnostic tests for 15 enteropathogens causing childhood diarrhoea: a multicentre study. Lancet Infect Dis. 2014; 14: 716–724. doi: 10.1016/S1473-3099(14)70808-4 25022434 10.1016/S1473-3099(14)70808-4

[17] Goldfarb DM, Steenhoff AP, Pernica JM, Chong S, Luinstra K, Mokomane M, et al Evaluation of anatomically designed flocked rectal swabs for molecular detection of enteric pathogens in children admitted to hospital with severe gastroenteritis in Botswana. J Clin Microbiol. 2014; 52: 3922–3927. doi: 10.1128/JCM.01894-14 25165077 10.1128/JCM.01894-14 PMC4313226

[18] Knudsen BE, Bergmark L, Munk P, Lukjancenko O, Priemé A, Aarestrup FM, et al Impact of sample type and DNA isolation procedure on genomic inference of microbiome composition. mSystems. 2016; 1: e00095–16. doi: 10.1128/mSystems.00095-16 27822556 10.1128/mSystems.00095-16 PMC5080404

[19] Wang WL, Xu SY, Ren ZG, Tao L, Jiang JW, Zheng SS. Application of metagenomics in the human gut microbiome. World J Gastroenterol. 2015; 21: 803–814. doi: 10.3748/wjg.v21.i3.803 25624713 10.3748/wjg.v21.i3.803 PMC4299332

[20] Boisen N, Scheutz F, Rasko DA, Redman JC, Persson S, Simon J, et al Genomic characterization of enteroaggregative *Escherichia coli* from children in Mali. J Infect Dis. 2012; 205: 431–444. doi: 10.1093/infdis/jir757 22184729 10.1093/infdis/jir757 PMC3256949

[21] Halligan E, Edgeworth J, Bisnauthsing K, Bible J, Cliff P, Aarons E, et al Multiplex molecular testing for management of infectious gastroenteritis in a hospital setting: a comparative diagnostic and clinical utility study. Clin Microbiol Infect. 2014; 20: 460–467. 10.1111/1469-0691.12476 24274687

