# Supplementary material for: Nonaplex PCR using Cliffhanger primers to identify diarrhoeagenic Escherichia coli from crude lysates of human faecal samples
Source: PLoS One. 2018 Jun 26;13(6):e0199766. doi: 10.1371/journal.pone.0199766 (PMC6019694; doi:10.1371/journal.pone.0199766)
Supplement: S1 Table — (DOC) [file pone.0199766.s001.doc]

**S1 Table.** Analysis of the Diarrhoeagenic *Escherichia coli* control strain collection by the routine method versus the Cliffhanger method.

| ***E. coli* strain collection from Statens Serum Institute** | | | |  | **Raw MFI by the Cliffhanger method for plate 1** | | | | | | | | |
| --- | --- | --- | --- | --- | --- | --- | --- | --- | --- | --- | --- | --- | --- |
| **Strain #** | Serotype | Group | Expected gene positivity | Routine method | *estAp* | *rrs* | *stx1* | *eae* | *aggR* | *estAh* | *elt* | *stx2* | *ipaH* |
| **NC** |  |  |  |  | 104 | 114 | 127 | 153 | 220 | 123 | 242 | 94 | 128 |
| **NC** |  |  |  |  | 84 | 92 | 166 | 100 | 192 | 105 | 178 | 73 | 109 |
| **NC** |  |  |  |  | 89 | 96 | 112 | 117 | 206 | 106 | 235 | 97 | 114 |
| **NC** |  |  |  |  | 77 | 88 | 87 | 95 | 190 | 97 | 89 | 71 | 102 |
| **NC** |  |  |  |  | 90 | 95 | 107 | 100 | 224 | 108 | 113 | 76 | 107 |
| **PC1** |  |  | *aggR, stx1, stx2, rrs* |  | 76 | 1705 | 1563 | 92 | 1011 | 95 | 85 | 2232 | 99 |
| **PC2** |  |  | *estAh, estAp, elt, rrs* |  | 1192 | 1637 | 88 | 107 | 130 | 624 | 2855 | 89 | 117 |
| **PC3** |  |  | *eae, ipaH, rrs* |  | 94 | 1824 | 113 | 1880 | 131 | 111 | 104 | 82 | 2955 |
| **fr 1368** | O172:H- | EIEC | *ipaH, rrs* | *ipaH, rrs* | 76 | 1883 | 92 | 99 | 152 | 97 | 90 | 68 | 3005 |
| **fr 1292** | O143:H- | EIEC | *ipaH, rrs* | *ipaH, rrs* | 86 | 1939 | 125 | 104 | 160 | 104 | 179 | 77 | 3135 |
| **fr 1294** | O143:H- | EIEC | *ipaH, rrs* | *ipaH, rrs* | 85 | 1944 | 96 | 102 | 168 | 106 | 106 | 74 | 3036 |
| **D2164** | O157:H7 | STEC | *stx1, stx2, eae, rrs* | *stx1, stx2, eae, rrs* | 88 | 1530 | 1460 | 1640 | 122 | 105 | 99 | 1967 | 103 |
| **D2166** | O78:K80:H11 | ETEC | *estAh, estAp, elt, rrs* | *estAh, elt, rrs* | 1237 | 1726 | 99 | 107 | 122 | 617 | 2871 | 86 | 110 |
| **D2168** | O6:K15 | ETEC | *estAh, estAp, elt, rrs* | *estAh, elt, rrs* | 98 | 1780 | 87 | 98 | 114 | 686 | 2826 | 79 | 106 |
| **D2190** | O21:K-H4 | DAEC | *rrs* | *rrs* | 83 | 1940 | 111 | 106 | 181 | 107 | 130 | 92 | 117 |
| **D2192** | O124:K-:H30 | EIEC | *ipaH, rrs* | *ipaH, rrs* | 88 | 1905 | 119 | 108 | 173 | 109 | 106 | 82 | 3143 |
| **D2194** | O9:K+:H4 | - | *rrs* | *rrs* | 82 | 1956 | 127 | 100 | 182 | 104 | 100 | 75 | 114 |
| **D2241** | Oru:K?:H25 | - | *rrs* | *rrs* | 80 | 1963 | 154 | 97 | 165 | 102 | 107 | 108 | 101 |
| **D2258** | O27:K?:H7 | ETEC | *estAp, rrs* | *rrs* | 1788 | 1902 | 100 | 98 | 126 | 103 | 92 | 75 | 109 |
| **D2259** | O115:K?:H5 | ETEC | *estAh,rrs* | *estAh,rrs* | 101 | 1791 | 104 | 96 | 123 | 691 | 94 | 75 | 104 |
| **D2260** | O8:K+:H9 | ETEC | *elt, rrs* | *elt, rrs* | 67 | 1827 | 79 | 82 | 113 | 88 | 3210 | 83 | 84 |
| **D2261** | O148:K-H28 | ETEC | *estAh, rrs* | *estAh, rrs* | 84 | 1825 | 85 | 89 | 108 | 800 | 86 | 107 | 94 |
| **D2263** | O27:K-:H20 | ETEC | *estAp, rrs* | *rrs* | 1718 | 1867 | 92 | 98 | 131 | 101 | 112 | 128 | 111 |
| **D2264** | O25:K+:H- | ETEC | *elt, rrs* | *elt, rrs* | 85 | 1887 | 95 | 104 | 135 | 107 | 3254 | 85 | 104 |
| **D2983** | O145:H- | STEC | *stx2, eae, rrs* | *stx2, eae, rrs* | 74 | 1656 | 83 | 1738 | 114 | 95 | 89 | 2106 | 95 |
| **D3271** | O145:H4 | STEC | *stx2, eae, rrs* | *stx2, eae, rrs* | 77 | 1724 | 83 | 1841 | 112 | 92 | 88 | 2117 | 96 |
| **D3295** | Oru:H- | ETEC | *elt, rrs* | *elt, rrs* | 83 | 1861 | 90 | 102 | 127 | 99 | 3286 | 79 | 104 |
| **D3296** | 021:H10 | DAEC | *rrs* | *rrs* | 79 | 1993 | 108 | 116 | 138 | 112 | 310 | 176 | 103 |
| **D3297** | O8:H9 | ETEC | *estAp, rrs* | *rrs* | 65 | 1979 | 94 | 79 | 180 | 86 | 92 | 126 | 85 |
| **D3299** | O17:Hru | ETEC | *estAp, elt, rrs* | *elt, rrs* | 1541 | 1850 | 88 | 98 | 120 | 98 | 3144 | 84 | 103 |
| **D3300** | O128ac:H+ | ETEC | *estAh, estAp, elt, rrs* | *estAh, elt, rrs* | 112 | 1796 | 91 | 98 | 118 | 574 | 2782 | 83 | 102 |
| **D3301** | O39:H12 | ETEC | *estAh, elt, rrs* | *estAh, elt, rrs* | 91 | 1687 | 76 | 86 | 106 | 639 | 2615 | 67 | 95 |
| **D3303** | O8:H9 | ETEC | *elt, rrs* | *elt, rrs* | 63 | 1839 | 82 | 80 | 107 | 85 | 3346 | 63 | 81 |
| **D3304** | O167:Hru | ETEC | *elt, rrs* | *elt, rrs* | 83 | 1860 | 92 | 95 | 128 | 102 | 3263 | 82 | 100 |
| **D3305** | O6:H16 | ETEC | *estAh, elt, rrs* | *estAh, elt, rrs* | 90 | 1807 | 86 | 82 | 99 | 658 | 2841 | 69 | 87 |
| **D3306** | O15:H- | DAEC | *rrs* | *rrs* | 62 | 1975 | 91 | 77 | 168 | 80 | 72 | 58 | 87 |
| **D3307** | O64:H- | EIEC | *ipaH, rrs* | *ipaH, rrs* | 81 | 1897 | 104 | 95 | 162 | 96 | 90 | 86 | 2965 |
| **D3308** | O64:H- | EIEC | *ipaH, rrs* | *ipaH, rrs* | 61 | 1900 | 77 | 81 | 162 | 80 | 70 | 61 | 2921 |
| **D3309** | O+:H- | EIEC | *ipaH, rrs* | *ipaH, rrs* | 76 | 1934 | 79 | 95 | 167 | 100 | 93 | 136 | 3109 |
| **D3310** | O121:H- | EIEC | *ipaH, rrs* | *ipaH, rrs* | 86 | 1952 | 107 | 99 | 179 | 104 | 100 | 154 | 3138 |
| **D3311** | O56:H- | ETEC | *elt, rrs* | *elt, rrs* | 80 | 1825 | 96 | 95 | 130 | 97 | 3318 | 154 | 104 |
| **D3312** | O28ac:H- | EIEC | *ipaH, rrs* | *ipaH, rrs* | 76 | 1920 | 105 | 101 | 145 | 103 | 130 | 73 | 2997 |
| **D3313** | O169:H- | ETEC | *elt, rrs* | *elt, rrs* | 77 | 1879 | 87 | 92 | 129 | 94 | 3268 | 79 | 96 |
| **D3314** | Oru:H- | ETEC | *estAh, elt, rrs* | *estAh, elt, rrs* | 104 | 1779 | 86 | 93 | 114 | 592 | 2656 | 76 | 95 |
| **D3315** | O114:H- | EPEC | *eae, rrs* | *eae, rrs* | 59 | 1811 | 76 | 1918 | 105 | 77 | 71 | 56 | 80 |
| **D3317** | O153:H2 | EAEC | *aggR, rrs* | *aggR, rrs* | 80 | 1823 | 91 | 94 | 1695 | 98 | 89 | 85 | 95 |
| **D3318** | O86:H8 | EPEC | *eae, rrs* | *eae, rrs* | 81 | 1870 | 89 | 1997 | 123 | 97 | 92 | 79 | 109 |
| **D3319** | O92:H33 | EAEC | *aggR, rrs* | *aggR, rrs* | 82 | 1908 | 97 | 95 | 1631 | 98 | 108 | 127 | 99 |
| **D3321** | O92:H31 | EAST1 | *rrs* | *rrs* | 80 | 1944 | 110 | 96 | 164 | 95 | 116 | 73 | 109 |
| **D3322** | O150:H28 | EAggEC | *rrs* | *rrs* | 78 | 1989 | 94 | 94 | 179 | 93 | 87 | 70 | 101 |
| **D3325** | O132:H34 | A/EEC | *eae, rrs* | *eae, rrs* | 80 | 1840 | 88 | 1987 | 119 | 93 | 85 | 74 | 99 |
| **D3326** | O173:H- | EIEC | *ipaH, rrs* | *ipaH, rrs* | 76 | 1966 | 91 | 89 | 158 | 94 | 88 | 78 | 3053 |
| **D3327** | O86:H8 | EPEC | *eae, rrs* | *eae, rrs* | 61 | 1876 | 66 | 2028 | 105 | 80 | 73 | 56 | 85 |
| **D3328** | O144:H- | EIEC | *ipaH, rrs* | *ipaH, rrs* | 79 | 1965 | 87 | 95 | 166 | 96 | 94 | 99 | 3055 |
| **D3329** | O+:H- | EIEC | *ipaH, rrs* | *ipaH, rrs* | 85 | 1960 | 101 | 104 | 190 | 103 | 96 | 84 | 3192 |
| **D3330** | O28ac:H- | EIEC | *ipaH, rrs* | *ipaH, rrs* | 92 | 1929 | 101 | 108 | 181 | 102 | 104 | 86 | 3072 |
| **D3331** | O148:H28 | ETEC | *estAp, elt, rrs* | *estAp, elt, rrs* | 1308 | 1856 | 86 | 97 | 123 | 103 | 3082 | 85 | 104 |
| **D3332** | O103:H2 | EPEC | *eae, rrs* | *eae, rrs* | 80 | 1863 | 92 | 1917 | 120 | 91 | 92 | 74 | 98 |
| **D3333** | O111:H- | EPEC | *eae, rrs* | *eae, rrs* | 75 | 1894 | 94 | 2069 | 119 | 93 | 90 | 89 | 93 |
| **D3334** | O26:H- | EPEC | *eae, rrs* | *eae, rrs* | 59 | 1829 | 97 | 1934 | 95 | 79 | 80 | 98 | 81 |
| **D3335** | O142:H34 | EPEC | *eae, rrs* | *eae, rrs* | 78 | 1847 | 86 | 1997 | 117 | 91 | 90 | 71 | 92 |
| **D3336** | O129:H11 | A/EEC | *eae, rrs* | *eae, rrs* | 72 | 1771 | 79 | 1836 | 117 | 91 | 89 | 71 | 94 |
| **D3337** | O111:H38 | A/EEC | *eae, rrs* | *eae, rrs* | 74 | 1890 | 91 | 1942 | 122 | 90 | 86 | 70 | 98 |
| **D3338** | O111:H9 | EPEC | *eae, rrs* | *eae, rrs* | 78 | 1892 | 84 | 2036 | 121 | 97 | 96 | 71 | 101 |
| **D3339** | O114:H49 | A/EEC | *eae, rrs* | *eae, rrs* | 77 | 1841 | 93 | 1978 | 115 | 88 | 82 | 77 | 91 |
| **D3340** | O145:H34 | A/EEC | *eae, rrs* | *eae, rrs* | 74 | 1852 | 79 | 1991 | 109 | 89 | 84 | 101 | 89 |
| **D3341** | O121:H19 | A/EEC | *eae, rrs* | *eae, rrs* | 73 | 1850 | 83 | 1955 | 113 | 90 | 94 | 127 | 90 |
| **D3342** | O126:H6 | A/EEC | *eae, rrs* | *eae, rrs* | 73 | 1915 | 78 | 2071 | 111 | 88 | 84 | 65 | 91 |
| **D3343** | O26:H- | EPEC | *eae, rrs* | *eae, rrs* | 61 | 1835 | 87 | 1969 | 105 | 79 | 76 | 75 | 81 |
| **D3344** | O55:H7 | EPEC | *eae, rrs* | *eae, rrs* | 66 | 1841 | 79 | 1991 | 112 | 87 | 86 | 64 | 95 |
| **D3345** | Oru:H8 | A/EEC | *eae, rrs* | *eae, rrs* | 60 | 1842 | 72 | 1899 | 101 | 77 | 69 | 54 | 80 |
| **D3346** | O125ab:H5 | A/EEC | *eae, rrs* | *eae, rrs* | 76 | 1878 | 87 | 2001 | 117 | 89 | 86 | 67 | 91 |
| **D3347** | O118:H8 | A/EEC | *eae, rrs* | *eae, rrs* | 60 | 1855 | 72 | 1952 | 102 | 74 | 70 | 58 | 80 |
| **D3348** | O35,O135:H1 | A/EEC | *eae, rrs* | *eae, rrs* | 77 | 1948 | 89 | 2017 | 116 | 95 | 90 | 69 | 93 |
| **D3349** | O127:H- | EPEC | *eae, rrs* | *eae, rrs* | 66 | 1849 | 82 | 2004 | 104 | 81 | 73 | 58 | 84 |
| **D3350** | O145:H- | EPEC | *eae, rrs* | *eae, rrs* | 78 | 1861 | 88 | 1934 | 117 | 90 | 95 | 67 | 95 |
| **D3351** | O51:H49 | A/EEC | *eae, rrs* | *eae, rrs* | 76 | 1873 | 138 | 2046 | 114 | 97 | 120 | 73 | 99 |
| **D3352** | Oru:H33 | A/EEC | *eae, rrs* | *eae, rrs* | 65 | 1882 | 74 | 2009 | 103 | 84 | 82 | 89 | 89 |
| **D3353** | O4,O123:H- | A/EEC | *eae, rrs* | *eae, rrs* | 77 | 1905 | 88 | 2024 | 122 | 92 | 88 | 71 | 102 |
| **D3354** | O145:H- | EPEC | *eae, rrs* | *eae, rrs* | 78 | 1854 | 85 | 1960 | 116 | 95 | 85 | 73 | 98 |
| **D3355** | O116:H+ | A/EEC | *eae, rrs* | *eae, rrs* | 64 | 1785 | 74 | 1938 | 103 | 81 | 75 | 66 | 83 |
| **D3356** | O26:H- | EPEC | *eae, rrs* | *eae, rrs* | 84 | 1803 | 101 | 1912 | 126 | 97 | 96 | 72 | 103 |
| **D3357** | O145:H- | EPEC | *eae, rrs* | *eae, rrs* | 73 | 1819 | 91 | 1973 | 116 | 90 | 90 | 64 | 93 |
| **D3358** | O157:H16 | EPEC | *eae, rrs* | *eae, rrs* | 62 | 1866 | 70 | 1993 | 105 | 78 | 71 | 54 | 80 |
| **D3359** | O177:H25 | A/EEC | *eae, rrs* | *eae, rrs* | 74 | 1862 | 103 | 1987 | 118 | 91 | 91 | 73 | 92 |
| **D3365** | O157:H7 | STEC | *stx1, stx2, eae, rrs* | *stx1, stx2, eae, rrs* | 64 | 1662 | 1511 | 1726 | 96 | 77 | 73 | 1769 | 78 |
| **D3366** | O157:H7 | STEC | *stx1, eae, rrs* | *stx1, eae, rrs* | 76 | 1761 | 1694 | 1852 | 117 | 96 | 91 | 69 | 106 |
| **D3369** | O157:H- | STEC | *stx1, eae, rrs* | *stx1, eae, rrs* | 60 | 1771 | 1740 | 1877 | 98 | 76 | 72 | 54 | 76 |
| **D3370** | O103:H2 | STEC | *stx2, eae, rrs* | *stx2, eae, rrs* | 82 | 1669 | 93 | 1758 | 120 | 99 | 95 | 1552 | 105 |
| **D3371** | O103:H2 | STEC | *stx2, eae, rrs* | *stx2, eae, rrs* | 81 | 1714 | 83 | 1810 | 118 | 96 | 88 | 1868 | 93 |
| **D3372** | O103:H2 | STEC | *stx2, eae, rrs* | *stx2, eae, rrs* | 72 | 1722 | 73 | 1812 | 107 | 91 | 86 | 2005 | 90 |
| **D3373** | O26:H11 | STEC | *stx2, eae, rrs* | *stx2, eae, rrs* | 86 | 1673 | 89 | 1757 | 127 | 106 | 98 | 2016 | 110 |
| **D3377** | O145:H+ | STEC | *stx1, eae, rrs* | *stx1, eae, rrs* | 89 | 1748 | 1764 | 1912 | 121 | 97 | 101 | 74 | 110 |
| **D3378** | O26:H- | STEC | *stx1, stx2, eae, rrs* | *stx1, stx2, eae, rrs* | 85 | 1568 | 1636 | 1647 | 119 | 100 | 93 | 1916 | 99 |
| **D3379** | O145:H28 | STEC | *stx1, eae, rrs* | *stx1, eae, rrs* | 69 | 1765 | 1736 | 1856 | 107 | 87 | 84 | 64 | 94 |
| **D3380** | O157:H- | STEC | *stx1, eae, rrs* | *stx1, eae, rrs* | 52 | 2120 | 1517 | 1638 | 110 | 78 | 276 | 72 | 703 |

MFI, median fluorescence intensity; NC, negative control; PC1-3, positive controls; EPEC, enteropathogenic *E. coli*; A/EEC, attaching and effacing *E. coli*; ETEC, enterotoxigenic *E. coli*; STEC, shiga toxin-producing *E. coli*; EIEC, enteroinvasive *E. coli*; EAEC, enteroaggregative *E. coli*; DAEC, diffuse adherence to cells *E. coli*. Yellow highlights are positive gene targets by the Cliffhanger method defined as a MFI above the mean MFI of the five negative controls plus three standard deviations. Green highlights are negative gene targets by the Cliffhanger method that according to the datasheet for the *E. coli* strain collection from Statens Serum Institute should have been positive for that gene.

| ***E. coli* strain collection from Statens Serum Institute** | | | |  | **Raw MFI by the Cliffhanger method for plate 2** | | | | | | | | |
| --- | --- | --- | --- | --- | --- | --- | --- | --- | --- | --- | --- | --- | --- |
| **Strain #** | Serotype | Group | Expected gene positivity | Routine method | *estAp* | *rrs* | *stx1* | *eae* | *aggR* | *estAh* | *elt* | *stx2* | *ipaH* |
| **NC** |  |  |  |  | 103 | 111 | 115 | 118 | 210 | 130 | 113 | 149 | 127 |
| **NC** |  |  |  |  | 84 | 96 | 99 | 101 | 211 | 104 | 98 | 76 | 107 |
| **NC** |  |  |  |  | 94 | 102 | 113 | 110 | 218 | 117 | 106 | 110 | 115 |
| **NC** |  |  |  |  | 76 | 89 | 83 | 101 | 188 | 109 | 88 | 68 | 100 |
| **NC** |  |  |  |  | 94 | 101 | 107 | 110 | 244 | 113 | 133 | 115 | 115 |
| **PC1** |  |  | *aggR, stx1, stx2, rrs* |  | 91 | 1689 | 1606 | 110 | 602 | 114 | 101 | 1874 | 113 |
| **PC2** |  |  | *estAh, estAp, elt, rrs* |  | 1171 | 1602 | 81 | 106 | 119 | 623 | 2677 | 80 | 108 |
| **PC3** |  |  | *eae, ipaH, rrs* |  | 82 | 1727 | 79 | 1733 | 125 | 104 | 93 | 72 | 2860 |
| **D3382** | O26:H- | STEC | *stx2, eae, rrs* | *stx2, eae, rrs* | 78 | 1449 | 89 | 1513 | 119 | 98 | 92 | 1511 | 106 |
| **D3383** | O26:H11 | STEC | *stx1, eae, rrs* | *stx1, eae, rrs* | 77 | 1669 | 1700 | 1739 | 113 | 94 | 88 | 64 | 97 |
| **D3385** | O145:H- | STEC | *stx2, eae, rrs* | *stx2, eae, rrs* | 86 | 1684 | 90 | 1775 | 129 | 107 | 102 | 2013 | 107 |
| **D3386** | O145:H- | STEC | *stx2, eae, rrs* | *stx2, eae, rrs* | 88 | 1688 | 90 | 1727 | 126 | 112 | 104 | 1943 | 114 |
| **D3387** | O157:H- | STEC | *stx1, stx2, eae, rrs* | *stx1, stx2, eae, rrs* | 78 | 1571 | 1597 | 1629 | 115 | 96 | 89 | 1736 | 102 |
| **D3388** | O157:H7 | STEC | *stx2, eae, rrs* | *stx2, eae, rrs* | 89 | 1571 | 93 | 1652 | 130 | 112 | 105 | 2224 | 111 |
| **D3390** | O157:H7 | STEC | *stx2, eae, rrs* | *stx2, eae, rrs* | 78 | 1702 | 80 | 1754 | 118 | 97 | 90 | 1968 | 98 |
| **D3392** | O157:H- | STEC | *stx1, stx2, eae, rrs* | *stx1, stx2, eae, rrs* | 93 | 1543 | 1570 | 1611 | 135 | 108 | 105 | 1595 | 114 |
| **D3393** | O157:H- | STEC | *stx1, stx2, eae, rrs* | *stx1, stx2, eae, rrs* | 88 | 1566 | 1575 | 1643 | 125 | 110 | 100 | 1698 | 108 |
| **D3394** | O26:H11 | STEC | *stx1, eae, rrs* | *stx1, eae, rrs* | 90 | 1677 | 1723 | 1788 | 123 | 104 | 101 | 75 | 111 |
| **D3395** | O26:H11 | STEC | *stx1, eae, rrs* | *stx1, eae, rrs* | 88 | 1686 | 1689 | 1781 | 122 | 103 | 100 | 75 | 108 |
| **D3396** | O157:H7 | STEC | *stx2, eae, rrs* | *eae, rrs* | 74 | 1783 | 84 | 1896 | 120 | 96 | 86 | 68 | 103 |
| **D3397** | O103:H2 | STEC | *stx1, eae, rrs* | *eae, rrs* | 69 | 1742 | 76 | 1809 | 112 | 90 | 79 | 61 | 97 |
| **D3398** | O157:H7 | STEC | *stx2, eae, rrs* | *stx2, eae, rrs* | 75 | 1682 | 77 | 1757 | 116 | 93 | 85 | 1972 | 97 |
| **D3400** | O26:H11 | STEC | *stx1, eae, rrs* | *stx1, eae, rrs* | 86 | 1586 | 1613 | 1736 | 120 | 105 | 94 | 85 | 109 |
| **D3402** | O103:Hru | STEC | *stx1, eae, rrs* | *stx1, eae, rrs* | 95 | 1656 | 1694 | 1732 | 135 | 114 | 110 | 81 | 119 |
| **D3408** | O157:H- | STEC | *stx2, eae, rrs* | *stx2, eae, rrs* | 92 | 1543 | 87 | 1639 | 130 | 114 | 102 | 2069 | 116 |

MFI, median fluorescence intensity; NC, negative control; PC1-3, positive controls; EPEC, enteropathogenic *E. coli*; A/EEC, attaching and effacing *E. coli*; ETEC, enterotoxigenic *E. coli*; STEC, shiga toxin-producing *E. coli*; EIEC, enteroinvasive *E. coli*; EAEC, enteroaggregative *E. coli*; DAEC, diffuse adherence to cells *E. coli*. Yellow highlights are positive gene targets by the Cliffhanger method defined as a MFI above the mean MFI of the five negative controls plus three standard deviations. Green highlights are negative gene targets by the Cliffhanger method that according to the datasheet for the *E. coli* strain collection from Statens Serum Institute should have been positive for that gene.

| ***E. coli* strain collection from Statens Serum Institute** | | | |  | **Raw MFI by the Cliffhanger method for plate 3**  **(rerun)** | | | | | | | | |
| --- | --- | --- | --- | --- | --- | --- | --- | --- | --- | --- | --- | --- | --- |
| **Strain #** | Serotype | Group | Expected gene positivity | Routine method | *estAp* | *rrs* | *stx1* | *eae* | *aggR* | *estAh* | *elt* | *stx2* | *ipaH* |
| **NC** |  |  |  |  | 115 | 127 | 134 | 131 | 159 | 134 | 161 | 160 | 139 |
| **NC** |  |  |  |  | 95 | 107 | 112 | 113 | 141 | 118 | 110 | 119 | 118 |
| **NC** |  |  |  |  | 85 | 92 | 97 | 108 | 122 | 102 | 96 | 73 | 107 |
| **NC** |  |  |  |  | 93 | 104 | 117 | 120 | 136 | 114 | 109 | 83 | 117 |
| **NC** |  |  |  |  | 91 | 102 | 113 | 108 | 135 | 111 | 99 | 79 | 109 |
| **PC1** |  |  | *aggR, stx1, stx2, rrs* |  | 72 | 1679 | 1610 | 96 | 756 | 99 | 89 | 1948 | 102 |
| **PC2** |  |  | *estAh, estAp, elt, rrs* |  | 1164 | 1594 | 97 | 118 | 135 | 633 | 2736 | 92 | 121 |
| **PC3** |  |  | *eae, ipaH, rrs* |  | 97 | 1741 | 100 | 1803 | 130 | 115 | 114 | 86 | 2759 |
| **D2168** | O6:K15 | ETEC | *estAh, estAp, elt, rrs* | *estAh, elt, rrs* | 90 | 1699 | 84 | 103 | 126 | 721 | 2783 | 120 | 106 |
| **D3296** | 021:H10 | DAEC | *Rrs* | *rrs* | 98 | 1880 | 109 | 125 | 136 | 122 | 107 | 104 | 125 |
| **D3297** | O8:H9 | ETEC | *estAp, rrs* | *rrs* | 78 | 1875 | 96 | 96 | 121 | 97 | 89 | 66 | 103 |
| **D3299** | O17:Hru | ETEC | *estAp, elt, rrs* | *elt, rrs* | 1625 | 1782 | 88 | 101 | 117 | 106 | 3092 | 81 | 107 |
| **D3300** | O128ac:H+ | ETEC | *estAh, estAp, elt, rrs* | *estAh, elt, rrs* | 110 | 1736 | 99 | 113 | 135 | 697 | 2932 | 140 | 119 |
| **D3310** | O121:H- | EIEC | *ipaH, rrs* | *ipaH, rrs* | 88 | 1890 | 107 | 111 | 128 | 107 | 101 | 152 | 2948 |
| **D3311** | O56:H- | ETEC | *elt, rrs* | *elt, rrs* | 91 | 1833 | 107 | 113 | 131 | 114 | 3333 | 90 | 114 |
| **D3380** | O157:H- | STEC | *stx1, eae, rrs* | *stx1, eae, rrs* | 102 | 1704 | 1779 | 1824 | 133 | 118 | 105 | 86 | 121 |
| **D3396** | O157:H7 | STEC | *stx2, eae, rrs* | *eae, rrs* | 95 | 1799 | 108 | 1950 | 132 | 113 | 103 | 117 | 117 |
| **D3397** | O103:H2 | STEC | *stx1, eae, rrs* | *eae, rrs* | 93 | 1763 | 109 | 1895 | 125 | 110 | 104 | 85 | 115 |

MFI, median fluorescence intensity; NC, negative control; PC1-3, positive controls; EPEC, enteropathogenic *E. coli*; A/EEC, attaching and effacing *E. coli*; ETEC, enterotoxigenic *E. coli*; STEC, shiga toxin-producing *E. coli*; EIEC, enteroinvasive *E. coli*; EAEC, enteroaggregative *E. coli*; DAEC, diffuse adherence to cells *E. coli*. Yellow highlights are positive gene targets by the Cliffhanger method defined as a MFI above the mean MFI of the five negative controls plus three standard deviations. Green highlights are negative gene targets by the Cliffhanger method that according to the datasheet for the *E. coli* strain collection from Statens Serum Institute should have been positive for that gene.
