# Supplementary material for: Nonaplex PCR using Cliffhanger primers to identify diarrhoeagenic Escherichia coli from crude lysates of human faecal samples
Source: PLoS One. 2018 Jun 26;13(6):e0199766. doi: 10.1371/journal.pone.0199766 (PMC6019694; doi:10.1371/journal.pone.0199766)
Supplement: S2 Table — (DOC) [file pone.0199766.s002.doc]

**S2 Table.** The Zip-sequences and MagPlex magnetic microspheres used in this study.

| Name | Oligonucleotide sequence | Amplicon | Bead # |
| --- | --- | --- | --- |
| ZIP025 | 5’-TCCGCAGTCCTGTTCCGCCA-Heg-CX-NH_2_ | *stx1* | 35 |
| ZIP037 | 5’-TTGGTTGGGAGGCTGCGGTG-Heg-CX-NH_2_ | *eae* | 37 |
| ZIP039 | 5’-CGTCGCTCTGGTTCGCTCGC-Heg-CX-NH_2_ | *estAh* | 46 |
| ZIP044 | 5’-GCCATCGCCCACAGCAGGAC-Heg-CX-NH_2_ | *estAp* | 27 |
| ZIP058 | 5’-GCGTTTGGTTGGCTGCGGAC-Heg-CX-NH_2_ | *rrs* | 29 |
| ZIP074 | 5’-TCCGCTGCCCACTTCCCTCG-Heg-CX-NH_2_ | *ipaH* | 62 |
| ZIP082 | 5’-CCACGCTCCGCTTTCCACCC-Heg-CX-NH_2_ | *aggR* | 44 |
| ZIP084 | 5’-AGCAACCCTCGCACCCTCGC-Heg-CX-NH_2_ | *elt* | 52 |
| ZIP088 | 5’-ACCCTCCGTCCGTTGGCGTC-Heg-CX-NH_2_ | *stx2* | 55 |

Heg, a hexaethylenglycol spacer; CX-NH_2_, an aminomodified cyclohexane spacer. Beads are Luminex® MagPlex®-C magnetic carboxylated microspheres.

**DNA oligonucleotides were coupled to** **MagPlex®-C magnetic carboxylated microspheres following the carbodiimide coupling procedure for amine-modified oligonucleotides as recommended by Luminex Corporation. In brief, 2.5×10^6^ microspheres were activated in 0.1 M MES, pH 4.5, followed by the addition of 0.2 nmol oligonucleotide and 25 µg EDC. The coupling reaction was incubated for 30 min in the dark, followed by the addition of 25 µg EDC and another 30 min incubation. 1.0 mL of 0.02% Tween-20 was added, and the supernatant was removed after magnetic separation for 1 min on a DynaMag™-2 magnetic particle concentrator (Invitrogen A/S, Tåstrup, Denmark). 1 mL of 0.1% SDS was added and vortexed, followed by magnetic separation and resuspension in 100 µL Tris-EDTA buffer, pH 8.0, and refrigerated storage.**
