# Supplementary material for: Nonaplex PCR using Cliffhanger primers to identify diarrhoeagenic Escherichia coli from crude lysates of human faecal samples
Source: PLoS One. 2018 Jun 26;13(6):e0199766. doi: 10.1371/journal.pone.0199766 (PMC6019694; doi:10.1371/journal.pone.0199766)
Supplement: S3 Table — (DOC) [file pone.0199766.s003.doc]

**S3 Table.** Two-by-two tables for the target genes shown in Fig 4.

| ***eae*** | | **Cliffhanger method** | |
| --- | --- | --- | --- |
|  |  | **Positive** | **Negative** |
| **Routine method** | **Positive** | **171** | **6** |
|  | **Negative** | **292** | **2046** |

Chi-squared test P < 0.0001

The 95% confidence interval for the difference between the proportions of samples that were positive by the Cliffhanger method versus by the routine method was between 10.1% and 12.6%

|  | | ***eae*** | |
| --- | --- | --- | --- |
|  |  | **Positive** | **Negative** |
| **Routine method** | **Positive** | **177** | **0** |
|  | **Negative** | **292** | **2046** |

Sensitivity: 37.7%

Specificity: 100.0%

Positive Predictive Value (PPV): 100.0%

Negative Predictive Value (NPV): 87.5%

|  | | ***eae*** | |
| --- | --- | --- | --- |
|  |  | **Positive** | **Negative** |
| **Cliffhan-ger method** | **Positive** | **463** | **0** |
|  | **Negative** | **6** | **2046** |

Sensitivity: 98.7% Specificity: 100.0%

PPV: 100.0% NPV: 99.7%

| ***stx1*** | | **Cliffhanger method** | |
| --- | --- | --- | --- |
|  |  | **Positive** | **Negative** |
| **Routine method** | **Positive** | **3** | **1** |
|  | **Negative** | **42** | **2469** |

P < 0.0001

95% confidence interval: 1.1% to 2.1%

|  | | ***stx1*** | |
| --- | --- | --- | --- |
|  |  | **Positive** | **Negative** |
| **Routine method** | **Positive** | **4** | **0** |
|  | **Negative** | **42** | **2469** |

Sensitivity: 8.7% Specificity: 100.0%

PPV: 100.0% NPV: 98.3%

|  | | ***stx1*** | |
| --- | --- | --- | --- |
|  |  | **Positive** | **Negative** |
| **Cliffhan-ger method** | **Positive** | **45** | **0** |
|  | **Negative** | **1** | **2469** |

Sensitivity: 97.8% Specificity: 100.0%

PPV: 100.0% NPV: 99.96%

| ***stx2*** | | **Cliffhanger method** | |
| --- | --- | --- | --- |
|  |  | **Positive** | **Negative** |
| **Routine method** | **Positive** | **7** | **0** |
|  | **Negative** | **43** | **2465** |

P < 0.0001

95% confidence interval: 1.2% to 2.2%

|  | | ***stx2*** | |
| --- | --- | --- | --- |
|  |  | **Positive** | **Negative** |
| **Routine method** | **Positive** | **7** | **0** |
|  | **Negative** | **43** | **2465** |

Sensitivity: 14.0% Specificity: 100.0%

PPV: 100.0% NPV: 98.3%

|  | | ***stx2*** | |
| --- | --- | --- | --- |
|  |  | **Positive** | **Negative** |
| **Cliffhan-ger method** | **Positive** | **50** | **0** |
|  | **Negative** | **0** | **2465** |

Sensitivity: 100.0% Specificity: 100.0%

PPV: 100.0% NPV: 100.0%

| ***elt*** | | **Cliffhanger method** | |
| --- | --- | --- | --- |
|  |  | **Positive** | **Negative** |
| **Routine method** | **Positive** | **17** | **0** |
|  | **Negative** | **81** | **2417** |

P < 0.0001

95% confidence interval: 2.5% to 3.9%

|  | | ***elt*** | |
| --- | --- | --- | --- |
|  |  | **Positive** | **Negative** |
| **Routine method** | **Positive** | **17** | **0** |
|  | **Negative** | **81** | **2417** |

Sensitivity: 17.3% Specificity: 100.0%

PPV: 100.0% NPV: 96.8%

|  | | ***elt*** | |
| --- | --- | --- | --- |
|  |  | **Positive** | **Negative** |
| **Cliffhan-ger method** | **Positive** | **98** | **0** |
|  | **Negative** | **0** | **2417** |

Sensitivity: 100.0% Specificity: 100.0%

PPV: 100.0% NPV: 100.0%

| ***estAh*** | | **Cliffhanger method** | |
| --- | --- | --- | --- |
|  |  | **Positive** | **Negative** |
| **Routine method** | **Positive** | **11** | **1** |
|  | **Negative** | **19** | **2484** |

P < 0.0001

95% confidence interval: 0.4% to 1.1%

|  | | ***estAh*** | |
| --- | --- | --- | --- |
|  |  | **Positive** | **Negative** |
| **Routine method** | **Positive** | **12** | **0** |
|  | **Negative** | **19** | **2484** |

Sensitivity: 38.7% Specificity: 100.0%

PPV: 100.0% NPV: 99.2%

|  | | ***estAh*** | |
| --- | --- | --- | --- |
|  |  | **Positive** | **Negative** |
| **Cliffhan-ger method** | **Positive** | **30** | **0** |
|  | **Negative** | **1** | **2484** |

Sensitivity: 96.8% Specificity: 100.0%

PPV: 100.0% NPV: 99.96%

| ***estAp*** | | **Cliffhanger method** | |
| --- | --- | --- | --- |
|  |  | **Positive** | **Negative** |
| **Routine method** | **Positive** | **0** | **0** |
|  | **Negative** | **26** | **2489** |

P < 0.0001

95% confidence interval: 0.6% to 1.4%

| ***ipaH*** | | **Cliffhanger method** | |
| --- | --- | --- | --- |
|  |  | **Positive** | **Negative** |
| **Routine method** | **Positive** | **9** | **1** |
|  | **Negative** | **38** | **2467** |

P < 0.0001

95% confidence interval: 1.0% to 2.0%

|  | | ***ipaH*** | |
| --- | --- | --- | --- |
|  |  | **Positive** | **Negative** |
| **Routine method** | **Positive** | **10** | **0** |
|  | **Negative** | **38** | **2467** |

Sensitivity: 20.8% Specificity: 100.0%

PPV: 100.0% NPV: 98.5%

|  | | ***ipaH*** | |
| --- | --- | --- | --- |
|  |  | **Positive** | **Negative** |
| **Cliffhan-ger method** | **Positive** | **47** | **0** |
|  | **Negative** | **1** | **2467** |

Sensitivity: 97.9% Specificity: 100.0%

PPV: 100.0% NPV: 99.96%

| ***aggR*** | | **Cliffhanger method** | |
| --- | --- | --- | --- |
|  |  | **Positive** | **Negative** |
| **Routine method** | **Positive** | **94** | **49** |
|  | **Negative** | **37** | **2335** |

P = 0.1971

95% confidence interval: -1.2% to 0.2%

|  | | ***aggR*** | |
| --- | --- | --- | --- |
|  |  | **Positive** | **Negative** |
| **Routine method** | **Positive** | **143** | **0** |
|  | **Negative** | **37** | **2335** |

Sensitivity: 79.4% Specificity: 100.0%

PPV: 100.0% NPV: 98.4%

|  | | ***aggR*** | |
| --- | --- | --- | --- |
|  |  | **Positive** | **Negative** |
| **Cliffhan-ger method** | **Positive** | **131** | **0** |
|  | **Negative** | **49** | **2335** |

Sensitivity: 72.8% Specificity: 100.0%

PPV: 100.0% NPV: 97.9%
